# Evaluation of a medical education policy with compulsory rural service in China

**DOI:** 10.3389/fpubh.2023.1042898

**Published:** 2023-02-02

**Authors:** Dan Hu, Baisong Zhang, Mingyu Huang, Min Liu, Xiulong Xia, Yanli Zuo, Xiaoyun Liu

**Affiliations:** ^1^China Center for Health Development Studies, Peking University, Beijing, China; ^2^Qinghai Center for Health Development Studies, Medical College of Qinghai University, Xining, Qinghai, China; ^3^Center for Rural Medical Education Research, Gannan Medical College, Ganzhou, Jiangxi, China; ^4^International Research Center for Rural Medical Education, Jiujiang University, Jiujiang, Jiangxi, China; ^5^Department of General Medicine, Guangxi Medical University, Nanning, China

**Keywords:** evaluation, China, policy, compulsory rural service, medical education

## Abstract

**Background:**

Since 2010, China has implemented a national programme to train general practitioners for rural areas. The programme enrolled medical students with a rural background who signed a contract for 6 years' compulsory rural service after graduation. China is transitioning its national COVID-19 strategies in view of the features of coronavirus Omicron variant, the vaccination coverage, and the need for socioeconomic development. Strengthening primary health care, especially the health workforce in rural areas, should be an important consideration during the policy transition. This study aims to evaluate the implementation process of enrolling medical students in the programme, their willingness to work in the rural settings and their actual job choice after graduation.

**Methods:**

The study chose four medical universities in central and western China. A total of 2,041 medical graduates who have signed a contract for compulsory rural service and 1,576 medical graduates enrolled “as usual” (no compulsory rural service) were recruited in five campaigns–every June from 2015 to 2019. A survey was conducted 1 week before their graduation ceremony.

**Results:**

The top three reasons for choosing this programme were: a recommendation of a family member or teacher, a guaranteed job after graduation and the waiver of the tuition fee. 23.0–29.7% of the study participants were not familiar with the policy details. 39.1% of the medical students signed a contract with a county other than that of their hometown. Medical graduates on the compulsory rural service programme had very low willingness (1.9%) to work in rural areas but 86.1% of them actually worked at township health centers. In contrast, the willingness to work at township health centers was 0.2% for the comparison group (medical graduates without the contract), and their actual job choice at township health centers was 0%.

**Conclusions:**

Although the well-trained medical graduates on the compulsory rural service programme have low willingness to work in the township health centers, 86.1% of them choose to do so following their contract. This programme will strengthen the primary health workforce to deal with the increasing disease burden as China is transitioning its national COVID-19 strategies.

## Introduction

Shortage of Human Resources for Health (HRH) in rural areas has long been a worldwide issue (1–3). In China, although the economy has experienced a rapid growth over the past three decades, the disparity of HRH between rural and urban areas has been enlarging (4). The need for well-trained doctors in rural areas is still an urgent challenge. Interventions to attract and retain rural health workers include medical education (e.g., enrolling students from rural areas), compulsory regulation, financial incentives, personal and professional support (5). Yet evaluations of the policy process and its effectiveness are very limited (6).

In 2010, along with the overall health system reform, China started a national medical education programme to train physicians for township health centers (THC) in central and western provinces. THC is the main type of PHC facilities in rural China. Although varying greatly in size, it usually has a team of 10–20 physicians working with other health workers to provide essential medical and public health services to a population of 20–50 thousand. Each year since 2010, 5,000 medical students with a rural background are enrolled into a 5-year free medical education programme and also granted a modest living allowance. They need to sign a contract with the medical university and local health authority, committing to work at THC for 6 years after graduation. Breaking the contract means repayment of the tuition fee plus a fine and constraints in medical practice within the province.

The COVID-19 pandemic starting in 2020 has posed additional challenges to the shortage of quality health professionals in rural PHC setting in China. During the COVID-19 pandemic, most PHC workers including the well-trained GP were intensively engaged in combating the pandemic, and therefore dedicated less efforts in maintaining the routine care to patients and communities (7). China is transitioning its national COVID-19 strategies and policies in consideration of the features of the Omicron variant, the vaccination coverage, and the need for economic development. There will be a surge in COVID-19 cases and pressures on health systems to meet the population's health needs. In this transitioning, rural primary health care will meet increasing challenges and pressures. Rural health systems in China are vulnerable for lack of well-trained general practitioners. Therefore, there is a pressing need to strengthen primary health workforce in rural area. Although the medical education programme with compulsory rural service in China was designed and implemented before the COVID-19 pandemic, it should be able to strengthen the rural workforce to support recovery from COVID-19.

The programme of training medical graduates committed to compulsory rural service has been implemented for 12 years. This study aimed to evaluate the programme implementation, including medical students' reasons for enrolling in the programme and their awareness of the details of the contract they have signed. The study also evaluated the programme effectiveness, in terms of the place of work of the newly qualified GPs and their current willingness to work in that place.

## Methods

### Study sites and participants

The study chose medical graduates from four medical universities in central and western provinces of China, Qinghai, University (QU), Jiujiang University (JU), Gannan Medical College (GMC), and Guangxi Medical University (GMU).

In each university, two groups of medical graduates were selected to participate in the study: one group of medical graduates who had signed a contract for compulsory rural service (hereafter referred to as intervention group), the other group of medical students enrolled “as usual” (without signing a contract for compulsory rural service, hereafter referred to as comparison group). They were recruited into the study in the summers from 2015 to 2019, 1 week before their graduation ceremony.

### Questionnaire

After providing informed consent, the graduates who agreed to participate in the study completed a questionnaire. Key literatures (5, 8) on interventions to attract and retain health professionals for rural areas were reviewed in order to develop the study design and questionnaire. We also consulted 14 experts including policy makers, medical education specialists, and researchers on human resources for health to provide advice on the questionnaire development. We did a pilot study with 50 participants in Qinghai University to further improve the questionnaire. We used the same questionnaire in all years to enable direct comparison of the findings.

The survey contents included questions about the graduates' demographic information, the reason for choosing the medical education programme with compulsory rural service (1 question), their knowledge about the programme (4 questions), and their willingness to work at THC after graduation (1 question). The majority of the questions were closed multiple choice questions. The questionnaires were administered in a classroom, where research team members were available to answer questions, if participants needed clarifications, and also checked all questionnaires for completeness when they were handed back in.

### Sampling

In all the four universities, the medical students who had signed a contract for compulsory rural service were enrolled into separate classes from other medical students. We included all medical graduates committed to compulsory rural service from the four universities and identified one class of medical students enrolled in the same year from the same university as a comparison group. The classes of the comparison group were consistently smaller in size than those of the intervention group. In total, 2041 students with compulsory contract and 1,576 students without compulsory contract were included in the study.

In addition to the questionnaire, we also conducted Focus Group Discussions (FGD) with medical students to explore their perception of the programme. Key informant interviews (KII) with teachers and programme managers were conducted to investigate the process of student admission, education, and deployment after graduation. Senior researchers from the research team conducted the FGDs as facilitator with postgraduate students as the observer and note takers. No other participants (e.g., university teachers or managers) were present during the FGDs. In each year, we conducted at least one focus group with a compulsory contract, KIIs with at least one teacher and one programme manager in each university (38 FGDs and 61 KIIs in total). All FGDs and KIIs were conducted in a meeting room or a classroom, and tape recorded after obtaining informed consents from the participants. Each FGD o KII lasted for 40 min to 1 h.

### Data analysis

The survey data were double entered into computer using Epidata 3.1. Descriptive analysis was applied to explore the characteristics of the medical students with compulsory rural service, including the reason for choosing the programme, their knowledge about the programme, the status of their contract, and their willingness to work at township health centers after graduation. Comparisons were made between the two groups with and without compulsory contract in terms of their willingness to work in THCs and their actual job choices after graduation.

Qualitative data were analyzed using the framework approach with the help of MaxQDA 10.0 (9). A thematic framework was developed based on the interview topic guides and emerging issues form the FGDs and KIIs. High level themes included reasons for choosing the medical education programme, student admission process, students' study motivation during the education process, deployment process after graduation, and perceptions about the programme.

## Results

Among the medical students with compulsory contract, 67.6% grew up in a rural setting while they were 0–15 years old. This was higher than the percentage for the comparison group (61.5%). The proportion of rural medical students varied from 56.6% in Jiujiang University to 79.1% in Gannan Medical University. Medical students with contracts were mostly from poorer families, having lower household annual income than the comparison group (Table 1).

**Table 1:** Demographic information of the study participants.

	**QU**	**GMU**	**JU**	**GMC**	**Total**
**Female medical graduates (** ***n*** **, %)**					
Intervention group	374 (56.2)	231 (48.3)	84 (38.2)	284 (42.1)	973 (47.7)
Comparison group	294 (61.9)	255 (56.2)	105 (40.4)	153 (40.1)	807 (51.4)
**Participants with rural background (** ***n*** **, %)**					
Intervention group	435 (65.5)	378 (79.1)	124 (56.6)	437 (65.1)	1,374 (67.6)
Comparison group	284 (59.8)	300 (66.2)	149 (57.8)	231 (60.5)	964 (61.5)
**Household annual income (Yuan)**					
Intervention group	35,904	30,800	47,540	40,622	37,450
Comparison group	48,572	40,857	55,396	54,306	48,810

1. There were 7, 17, and 234 missing data points in responses to the three questions on gender, rural background and household income; 2. The “total” column is the sum total or average of participants from the four universities.

The top reason for choosing the programme was a recommendation from their family member or a teacher (28.7%). The second and third reasons were guaranteed job after graduation (22.1%) and free tuition (19.2%). Some variations could be found among the four universities. For example, more students in Qinghai University (which is located in one of the poorest provinces in China) chose the programme because it can guarantee a job after graduation (Table 2).

**Table 2:** Top reason for choosing the medical education programme (*n*, %).

	**QU**	**GMU**	**JU**	**GMC**	**Total**
Guaranteed job after graduation	245 (37.0)	36 (7.5)	44 (20.2)	124 (18.3)	449 (22.1)
Low score in college entrance exam	20 (3.0)	121 (25.3)	50 (22.9)	167 (24.7)	358 (17.6)
Free tuition	94 (14.2)	164 (34.3)	31 (14.2)	101 (14.9)	390 (19.2)
Family member or teacher recommendation	231 (34.8)	111 (23.2)	51 (23.4)	192 (28.4)	585 (28.7)
Permanent job position	32 (4.8)	4 (0.8)	15 (6.9)	25 (3.7)	76 (3.7)
Work location close to home	5 (0.8)	12 (2.5)	9 (4.1)	14 (2.1)	40 (2.0)
Reasonable income	11 (1.7)	3 (0.6)	1 (0.5)	5 (0.7)	20 (1.0)
Can get the work experiences from grass-roots service	5 (0.8)	6 (1.3)	6 (2.8)	7 (1.0)	24 (1.2)

1. Participants were asked to provide up to three reasons for choosing the programme in order of priority. This table shows the results for the first reason. 2. There were 7 missing data points for this question; 3. Low score in college entrance exam means they would have no chances to be admitted to medical school under normal conditions.

23.0% of medical graduates with a compulsory service contract did not have detailed information about the medical education programme, implying they were uncertain how the policy would work in practice. Large variations were found among the four universities. Students from Guangxi Medical University had better knowledge about the programme, while in Qinghai University, more than half of the students did not know details about their contract with the local health bureau and the university (Table 3).

**Table 3:** Percentage of the students who did not know the policy details (*n*, %).

**Policy details**	**QU**	**GMU**	**JU**	**GMC**	**Total**
No tuition fee, living allowance	165 (24.8)	57 (11.9)	60 (27.4)	187 (27.6)	469 (23.0)
Contract with local health bureau and university	352 (53.1)	55 (11.6)	30 (13.9)	107 (15.9)	544 (26.8)
6-year service at THC	299 (45.0)	37 (7.8)	66 (30.4)	202 (29.9)	604 (29.7)
Repay and fine if breaking contract	279 (42.0)	53 (11.1)	38 (17.4)	108 (16.0)	478 (23.5)

There were 1, 11, 7 and 7 missing data points in the four questions respectively.

The medical education programme encouraged participants to sign a contract with their hometown so that they are more likely to fulfill it and go back to their hometowns to work after graduation. However, 60.9% student did so, the other 39.1% students signed a contract with a county other than that of their hometown. Almost all (97.6%) students in Guangxi have signed a contract with their hometown, while in Qinghai only 24.9% of students did so (Table 4).

**Table 4:** Percentage of the graduates' willingness to work and actual work at THC.

	**QU**	**GMU**	**JU**	**GMC**	**Total**
Proportion of signed contracts with hometown	158 (24.9)	446 (97.6)	118 (57.6)	460 (71.3)	1,182 (60.9)
**Willing to work at THC**					
Intervention group	10 (1.5)	18 (3.8)	2 (0.9)	8 (1.2)	38 (1.9)
Comparison group	2 (0.4)	0	0	1 (0.3)	3 (0.2)
**Actual work at THC**					
Intervention group	619 (92.9)	386 (80.8)	175 (79.5)	578 (85.4)	1,758 (86.1)
Comparison group	0	0	0	0	0

There were 100 missing data points for the hometown contract question, and 41 missing data points for the willingness question.

Qualitative data revealed that the reason for the variation was the use of different criteria and processes for student admission across the four universities. In Qinghai, students with better performance in the college entrance examination were allowed to choose their favorite county to sign a contract with. They usually chose rural counties close to an urban city with better socioeconomic development. Those with lower examination performance had to sign a contract with a less developed county. This arrangement resulted in a high proportion of students not signing a contract with their hometown. The process was quite different in Guangxi, where the enrolled medical students can only sign a contract with their hometown. The other two universities (Jiujiang and Gannan) applied similar admission policy as in Guangxi, but when there were not enough eligible candidates in a county, they could recruit applicants from neighboring counties.

Only 1.9% of medical graduates in the intervention group reported willingness to work at THC, yet 86.1% actually worked at THC. In contrast, the willingness to work at THC was 0.2% for the comparison group, and the actual job choice at THC was 0 (Table 4).

## Discussion

This study analyzed the implementation process and initial effectiveness of a medical education programme with compulsory rural service in China. It found that the programme recruited students from poor rural families. The students' knowledge about the programme was quite limited. Some students did not sign contracts with their hometown because of different student admission procedures. Although the students had very low willingness to work at THC, the majority of them followed their contract to work at THC after graduation.

The medical education programme with compulsory rural service in China has been designed based on international experiences. First, medical students are recruited from rural backgrounds so that they are more likely to return to the countryside after graduation (10–12). A systematic review by Grobler et al. (8) showed that “rural origin is the single factor most strongly associated with rural practice.” Second, courses about rural health and internships in rural health facilities are built into the curriculum of this special medical education programme in order for medical graduates to appropriate knowledge and ability to work in rural areas. All the four medical universities made considerable efforts in developing the rural courses. Including courses about rural health and internships in rural health facilities have been proved to be effective interventions (5, 13, 14). Finally, financial incentives and compulsory regulations are combined to attract and retain rural health workers. Free medical education plus living allowances are the financial incentives, while 6 years' rural service after graduation is the compulsory regulation. A similar programme in Japan had positive results in staffing rural health service (15–17).

However, our study found some significant barriers in the implementation process. First, the students had limited knowledge about the programme. The two main reasons for choosing the programme were the free tuition and guaranteed job position after graduation, but some students did not have proper knowledge about their commitment to rural service. This indicates the policy needs to be further disseminated to the target candidates. Only with full understanding of the advantages and disadvantages of the policy, the programme will identify and recruit suitable candidates who have high willingness to work at THC. Second, due to different admission processes, some students, especially those in Qinghai University, did not sign a contract with their hometown county. Local recruitment and local deployment have been well-documented as an effective measure to attract and retain rural health workers (18–20). Local education authorities need to improve the student admission process by applying the principle of “local recruitment and local deployment.”

The study also found medical students had a very low willingness to work at THCs. This is understandable since rural health workers in China have a very low income level and limited career development opportunities, compared to their counterparts in urban areas (4). However, the survey showed that most of the students adhered to their contract to work at THCs. This is most likely driven by the compulsory measures. If the students break the contract, they should return the tuition fee, and the local health authority will also prohibit their medical practice in the region. The low willingness to work at THC is a serious concern for the sustainability of the programme. One of the key constraints is the selection and recruitment process. Candidates are not well-informed of the implementation of the programme. They choose the programme because of the guaranteed job, and free medical education, without even exactly understanding the conditions of the contract, i.e., 6 years' rural service. Only by the end of the 5-year medical education when the survey was conducted, they begin to understand the constraints they are facing. The selection and recruitment process should disseminate far more effectively the policy design and implementation details, to select and recruit the potential candidates who have enthusiasm to serve the rural population, or who may have higher willingness to fulfill the contract to work in rural areas.

In addition, financial and non-financial incentives should be developed to improve the medical graduates' willingness to work for rural primary health care. Salary increase, rural allowances, and performance-based awards are possible options to increase the attractiveness of rural job positions and to increase medical graduates' willingness to serve the rural population. Career development opportunities including in-service training, promotion of professional titles and management positions, and short-term work opportunities in higher level hospitals are potential non-financial incentives to increase their willingness. There is no single intervention that can work as magic bullet to address the shortage of health professionals in rural areas. By combining multiple measures, including selection of appropriate medical student candidates, free medical education, compulsory rural service contract, financial and non-financial incentives, it is possible to attract more medical graduates to work in the rural areas.

Cheng et al. (7) showed that during the COVID-19 pandemic, medical graduates from this special medical education programme were intensively involved in the pandemic response, including health promotion and education, community epidemic prevention and management, and nucleic acid testing and screening, among others. Although they faced challenges including lack of protective equipment and worries about themselves and their family being infected, more than half of them reported an increase in daily clinical workload, implying their important role in maintaining routine care during the pandemic. During the current COVI-19 strategy transition, China has encouraged more PHC facilities to provide medical services to the increasing number of patients infected with COVID-19 (21). The GPs trained in this programme will play an important role in the post-COVID recovery of the health system.

While these findings may illustrate the importance of this medical education programme with compulsory rural service in supporting the health system recovery from COVID-19, there are two important limitations of the programme to be further improved. First, the low willingness to work at THCs may predict high attrition rate after they complete the 6 years' contract as analyzed previously. This will reduce the potential of this medical education programme in supporting responses to future public health emergencies like COVID-19. Second, the curricula for this special education programme were designed far before the COVID-19 pandemic, with a focus on medical science and less on public health issues. Considerable efforts should be made to improve the curricula design to achieve goals of responding to disruptive public health events and increasing health system resilience.

The study collected data for 5 cohorts of medical graduates, forming the biggest cohort to investigate the implementation process and effectiveness of the medical education programme in China. The study has a few limitations. First, data for this paper was mainly from the baseline survey of the cohort study. The actual job choice of the medical graduates after they complete the contract at THCs cannot be measured at this stage. Second, the questionnaire used for the study was developed by the research team without rigorous analysis of its validity and reliability.

The preliminary evaluation of China's medical education programme with compulsory rural service shows positive results and potential in supporting health system resilience and recovery from COVD-19. The cohort will be further followed to investigate the long-term effects of the medical education programme in attracting and retaining health workers for rural China.

## Data availability statement

The original contributions presented in the study are included in the article/supplementary material, further inquiries can be directed to the corresponding author.

## Ethics statement

The studies involving human participants were reviewed and approved by the Ethics Committee of Peking University Health Sciences Center. The patients/participants provided their written informed consent to participate in this study.

## Author contributions

XL and DH contributed to the original conception and design of the study, interpretation of the data, and final revision of the manuscript. BZ contributed to data collection and analysis. MH, ML, XX, and YZ contributed to field work coordination, data interpretation, and revision of the manuscript. All authors read and approved the final manuscript.
